# Supplementary material for: The interactome of intact mitochondria by cross-linking mass spectrometry provides evidence for coexisting respiratory supercomplexes
Source: Mol Cell Proteomics. 2017 Dec 8;17(2):216–32. doi: 10.1074/mcp.RA117.000470 (PMC5795388; doi:10.1074/mcp.RA117.000470)
Supplement: Supplemental Data [file supp_RA117.000470_133922_0_supp_23978_fzffwf.zip › spectra_annotation/mito_DR_spectra_annotation/499-1-11-1-8-1.pdf]

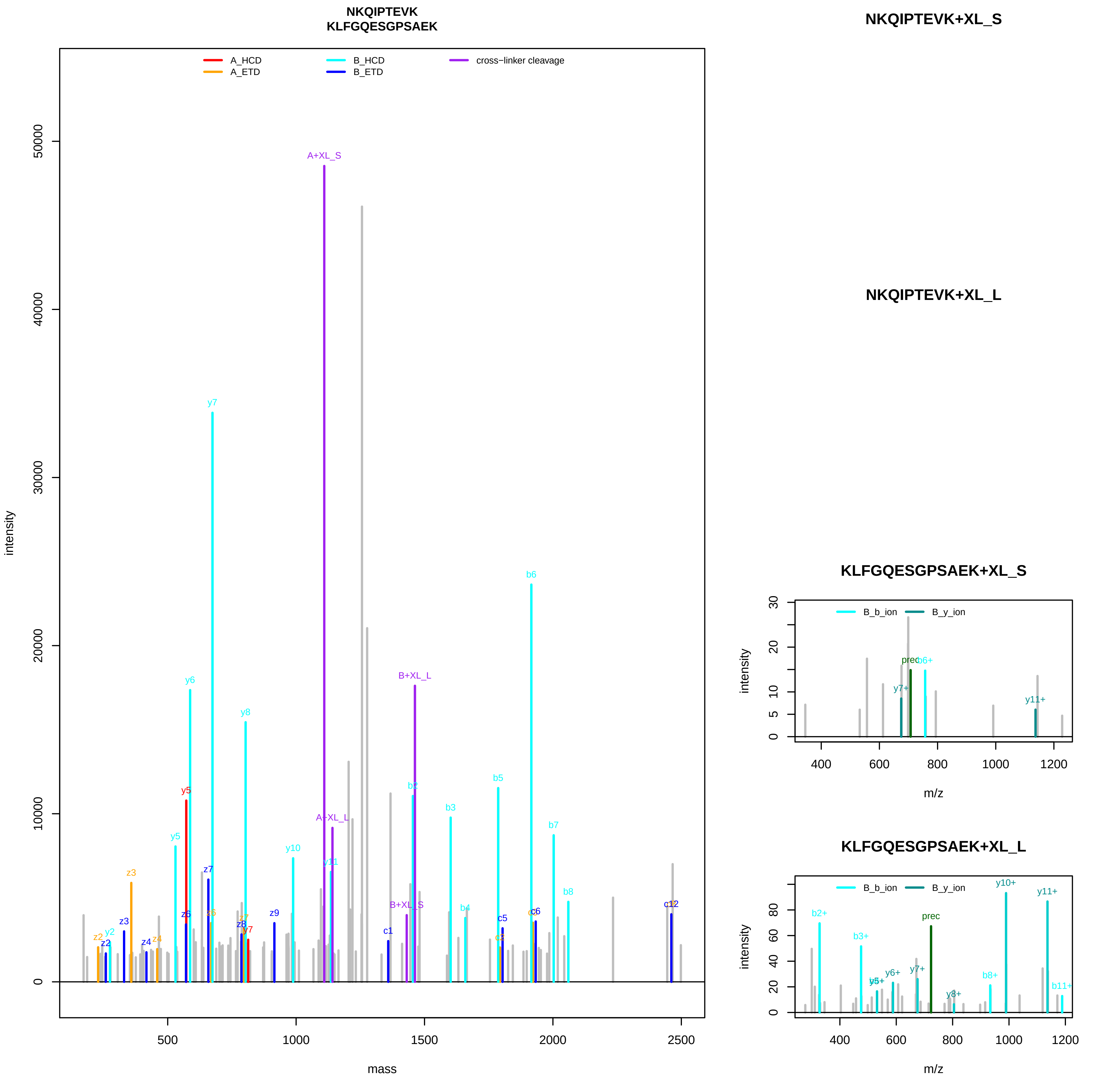
NKQIPTEVK
KLFGQESGPSAEK
A_HCD
A_ETD
B_HCD
B_ETD
cross−linker cleavage
A+XL_S
B+XL_L
A+XL_L
B+XL_S
y2 y5 y6 y7 y8 y10 y11
z2 z3 z4 z6 z7 z8 z9
y5 y7
b2 b3 b4 b5 b6 b7 b8
c1 c5 c6 c12
intensity
mass
0
10000
20000
30000
40000
50000
500
1000
1500
2000
2500
NKQIPTEVK+XL_S
NKQIPTEVK+XL_L
KLFGQESGPSAEK+XL_S
B_b_ion
B_y_ion
prec
b6+
y7+
y11+
intensity
m/z
0
5
10
20
30
400
600
800
1000
1200
KLFGQESGPSAEK+XL_L
B_b_ion
B_y_ion
b2+
b3+
y5+
y6+
y7+
y8+
b8+
y10+
y11+
b11+
prec
intensity
m/z
0
20
40
60
80
400
600
800
1000
1200
